# Highly Efficient Cellulose Nanofiber/Halloysite Nanotube Separators for Sodium-Ion Batteries

**DOI:** 10.3390/nano15221745

**Published:** 2025-11-20

**Authors:** Jiangwei Li, Qian Guan, Hualiang Wei, Mengju Zhang, Suxia Ren, Lili Dong, Zaifeng Li, Shuhua Yang, Xiuqiang Zhang

**Affiliations:** 1 Institute of Energy Co., Ltd., Henan Academy of Sciences, Zhengzhou 450008, China; 2 Key Biomass Energy Laboratory of Henan Province, Zhengzhou 450008, China; 3 Quality Inspection and Analysis Testing Research Center, Henan Academy of Sciences, Zhengzhou 450008, China; 4 Institute of Urban and Rural Mining, Changzhou University, Changzhou 213164, China

**Keywords:** separator, sodium-ion battery, cellulose nanofiber, halloysite nanotube

## Abstract

As a fundamental component of sodium-ion batteries, separators are considered to isolate two electrodes and simultaneously allow for the transport of ions. Cellulose separators have attracted widespread interest for their remarkable properties. In this study, we prepared composite separators comprising cellulose nanofibers (CNFs) and halloysite nanotubes (HNTs) for sodium-ion batteries. When the content of the HNT was up to 60%, the tensile strength and elongation at break of the composite separator (denoted as C/H-60) were 24.39 MPa and 2.22%, respectively. Importantly, the C/H-60 separator demonstrated a high porosity (69.08%), improved ionic conductivity (1.142 mS/cm), decent thermal stability, and good electrolyte retention (91.3% electrolyte uptake). The assembled sodium-ion battery containing the composite separators had an excellent rate capacity and cycling property. The proposed composite separators are expected to be applied in high-performance sodium-ion batteries.

## 1. Introduction

Energy is fundamental to the success and development of human society. A key feature of future energy solutions, for example, wind, solar, and tidal energy, is the storage of energy through batteries [1]. Over the past few decades, lithium-ion batteries have been widely used in electric vehicles, portable electronic devices, solar energy storage systems, and communications equipment [2,3]. Lithium-ion batteries have the advantages of high energy density, good cycle performance, and no memory effect [4,5,6]. However, the scarcity of lithium resources and high cost limit their application [7]. Therefore, sodium-ion batteries have received increased attention due to the abundance of sodium resources and similar electrochemical characteristics to lithium-ion batteries [7,8,9].

The separator is an important component of the sodium-ion battery, which not only avoids direct contact between the positive and negative electrodes, but also holds the electrolyte to facilitate the shuttle movement of ions inside the battery [10,11]. The separator determines the interface structure and internal resistance of the battery, which directly affects battery characteristics such as capacity, cycling, and safety performance [12]. However, there is limited research on separators for sodium-ion batteries. At present, the materials used in sodium-ion batteries are mostly polyolefins, such as polypropylene and polyethylene [13]. Regrettably, these separators have inherent defects, including poor electrolyte wettability and low thermal stability, which not only lead to high interfacial internal resistance but also may cause short-circuiting of the battery, or even fires and explosions [14]. Inorganic fiberglass separators are considered another choice [15]. Unfortunately, the mechanical strength of the glass fiber separator is poor, which makes it unsuitable for battery assembly in mass production [1]. Hence, it is urgent to prepare a new type of separator suitable for sodium-ion batteries.

Cellulose is an abundant biomass material with good strength and thermal stability, and its inherent hydroxyl group gives cellulose excellent wettability and chemical modification potential [16,17,18]. CNFs are nanoscale materials with high specific surface area and aspect ratio, isolated from raw materials. CNF can be made into a porous separator with a three-dimensional network structure and good strength and chemical stability by an appropriate process, which makes it an ideal material for the preparation of high-performance sodium-ion battery separators. Sheng et al. [19] successfully prepared a nanocellulose porous separator with high porosity, uniform nanopore structure, and excellent mechanical properties. After heat treatment, no significant dimensional changes were observed in the separator. Liu et al. [20] designed a porous separator based on the hydrogen bonding effect between CNF and lignosulphonate. The composite separators exhibited high porosity, excellent thermal stability, and remarkable electrolyte wettability. Liu et al. [21] prepared polyformaldehyde/CNF blend separators showing considerable tensile strength. Owing to the abundant polar groups and highly porous structure, the separators possessed high electrolyte uptake compared with the commercial polyethylene separators.

**Figure 1:**
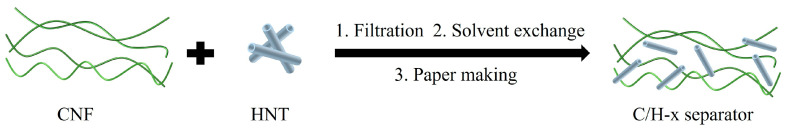
The preparation of CNF/HNT composite separators.

Most CNF-based separators are prepared from aqueous suspensions. During the drying process, strong capillary interactions between the fibers lead to the collapse of the pore structure, resulting in the dense structure of the separators [22]. This pore structure negatively affects the electrochemical performance of the battery. The incorporation of nanoscale fillers contributes to solving this problem. HNTs are multi-walled hollow tubular structures composed of two layers of aluminosilicate minerals. The outer layer of HNTs consists of silica tetrahedra, while the inner layer comprises alumina octahedra. Their inner surface exhibits properties similar to Al_2_O_3_, with chemical characteristics akin to SiO_2_. Compared to other ceramics/nanotubes, HNTs feature active groups that promote interactions with guest molecules due to their positive inner surface and negative outer surface. Furthermore, HNTs exhibit a high aspect ratio, large surface area, excellent tensile strength, good biocompatibility, fire resistance, and ease of chemical modification, making them a promising material in the battery field [23,24]. In this paper, we prepared a series of composite separators suitable for sodium-ion batteries. The separators consisting of CNF and HNT were formed through vacuum filtration, solvent change, and paper-making processes (Figure 1). The structural, physical, and electrochemical properties of the separators were further studied.

## 2. Materials and Methods

### 2.1. Materials

TEMPO-oxidized CNFs (1.53 wt%) were provided by Henan Xinrongyuan Co., Ltd. (Jiaozuo, China). HNTs were purchased from XFNANO Material Technology Co., Ltd. (Nanjing, China). Anhydrous ethanol was obtained from Shanghai Aladdin Reagent Co., Ltd. (Shanghai, China). Sodium-ion electrolyte (1M NaPF_6_ in Diglyme = 100 Vol%), sodium nanosheet (12 mm in diameter), 2032-type coin cell, and Na_3_V_2_(PO_4_)_3_ were used from DodoChem (Suzhou, China). Glass fiber (GFA) separators were bought from Whatman (Maidstone, UK). Distilled water was used in all experiments.

### 2.2. Preparation of CNF/HNT Separators

Different amounts of HNT were added to the CNFs suspension while guaranteeing that the total mass of both was 80 mg, where the mass ratios of HNT were 0%, 15%, 30%, 45% and 60%. After vigorous stirring for 2 h to disperse the mixtures and sonication for 5 min to reduce bubbles formed during the stirring process, the mixtures were vacuum filtered to produce the films. The films were then soaked in anhydrous ethanol for 12 h to extract all water, followed by hot pressing and vacuum drying at 90 °C for 90 s using a paper machine. The obtained samples were labeled as C/H-0, C/H-15, C/H-30, C/H-45, and C/H-60, respectively.

### 2.3. Characterization

The morphologies of the samples were characterized by scanning electron microscope (SEM, GeminiSEM 360, Oberkochen, Germany). The mechanical properties of all separators prepared into several dumbbell-type test splines (4 mm × 50 mm) by a specimen slicer were examined using an electronic universal testing machine (AGS-100, SHIMADZU, Kyoto, Japan) at a tensile speed of 1 mm/s. The thermal stabilities of the separators were measured through a thermogravimetric analyzer (TG, SDT Q600, TA Instrument, Milford, MA, USA) under a nitrogen atmosphere from 25 to 700 °C at a heating rate of 10 °C/min. Contact angle measurement was performed to investigate the electrolyte wettability of all separators by a contact angle meter (DSA25, KRÜSS, Hamburg, Germany).

The dry separators (15.8 mm in diameter) were soaked in the electrolyte for 2 h. The electrolyte uptake was calculated by Equation (1).

(1) $$Electrolyte\;uptake\left(\%\right)=\frac{m-m_{0}}{m_{0}}\times 100\%$$

where *m*_0_ and *m* were the mass of the separators before and after electrolyte soaking, respectively. The porosity of the separators was determined by an n-butanol absorption approach [25] and calculated by Equation (2).

(2) $$Porosity\left(\%\right)=\frac{m-m_{0}}{\rho \times V}\times 100\%$$

where *m*_0_ and *m* were the mass of the separators before and after immersion in the n-butanol; *ρ* and *V* were the density of the n-butanol and volume of the separators, respectively. Average values were recorded based on at least three independent experiments.

The electrochemical workstation (CHI660E, CH Instruments, Shanghai, China) was used to test electrochemical impedance spectroscopy (EIS) with the scanning frequency range of 0.1 to 10^5^ Hz and the amplitude of 5 mV, and linear sweep voltammetry (LSV) with the voltage range from 2.5 to 5.5 V at the scanning rate of 0.002 v/s. The calculation of Formula (3) for ionic conductivity (*σ*) was as follows:

(3) $$\sigma =\frac{d}{R_{b}\times S}$$

where *d* was the separator thickness, *R_b_* was the bulk resistance, and *S* was the effective area of the separator.

The electrochemical performance of separators was investigated through 2032-type coin cells assembled by sandwiching the separator between Na_3_V_2_(PO_4_)_3_ cathode and Na anode, and performed on the LAND battery testing system (CT2001A, Wuhan Land Electronics, Wuhan, China) in the voltage range 2.5–4.2 V.

## 3. Results and Discussion

### 3.1. Preparation of Composite Separators

**Figure 2:**
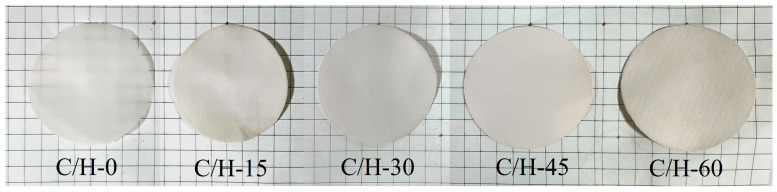
Photographs of C/H-x composite separators.

In this study, the composite separators were prepared via the Filtration–Ethanol solvent exchange–Paper making method, and photographs are shown in Figure 2. It could be clearly seen that the surfaces of the separators were smooth. The thickness of the separators increased from 0.078 mm (C/H-0) to 0.108 mm (C/H-45) with increasing HNT addition, as shown in Table 1. It should be noted that when the HNT content exceeded 60%, the CNF fraction became insufficient to form a self-standing separator. This deficiency weakened the HNT-CNF interactions, resulting in a lack of the structural strength and cohesion required for a robust separator.

It is well known that the separators should have high porosity to store enough liquid electrolyte to provide channels for a sodium ion, thus improving the charging and discharging performance [26]. CNF films formed in water comprise a dense network containing very small holes, causing a decrease in porosity, which hinders their use in batteries [27]. Thus, the ethanol solvent exchange approach was used to weaken this negative effect. During the immersion process, ethanol replaced the water between the cellulose fibers, which not only made the fibers looser and more porous, but also made the obtained film less prone to shrinkage during the subsequent drying process. Furthermore, because of the difference in surface tension between ethanol and water, ethanol served as a separating agent to enable the wet CNF films to be easily peeled off from the filter membrane, which allowed the films to be soaked individually and avoided the effect of simultaneous soaking [19]. In addition, the increase in HNTs also improved the porosity, wherein the C/H-60 separator had a porosity of 69.08% (Table 1). This was due to the fact that the incorporation of high specific surface area HNTs disturbed the orderly arrangement of nanofibers during the film formation process, thereby increasing the porosity.

### 3.2. Morphology Study of Composite Separators

**Figure 3:**
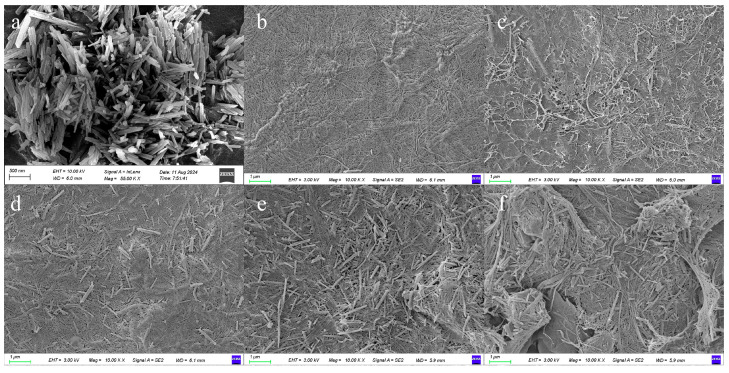
SEM images of HNT and C/H-x composite separators. (**a**) HNT, (**b**) C/H-0, (**c**) C/H-15, (**d**) C/H-30, (**e**) C/H-45, and (**f**) C/H-60.

The morphology of the samples was investigated using SEM (Figure 3). As shown in Figure 3a, the HNTs exhibited a typical tubular structure; the surface of the C/H-0 separator presented an obvious fiber structure, which was owing to the tight stacking of CNFs (Figure 3b). With the addition of HNTs, a microporous structure appeared on the film surface (Figure 3d). As the content of HNT further increased, more microporous structures emerged in the separator (Figure 3e). However, the separator showed delamination in Figure 3f, which was due to the low content of CNF as the backbone material. SEM image results were consistent with the porosity data (Table 1), indicating that the HNT could effectively adjust the porosity of the separator and thus improve its electrochemical performance.

### 3.3. Wettability of Composite Separators

**Figure 4:**
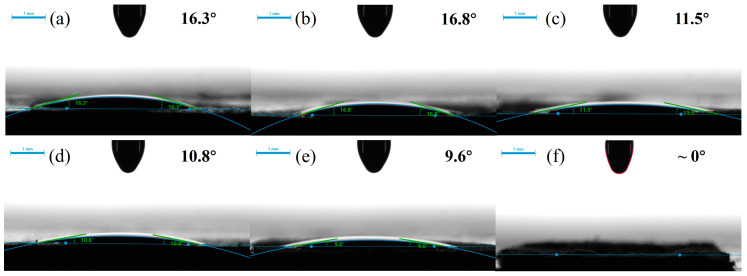
Photos of contact angle of separators. (**a**) C/H-0; (**b**) C/H-15; (**c**) C/H-30; (**d**) C/H-45; (**e**) C/H-60; (**f**) GFA.

The affinity between the electrolyte and separator has a significant effect on the performance of the batteries. High affinity can be obtained by increasing the wettability of the separators, which also makes the high loading of electrolyte possible. Higher wettability expands the contact surface between the electrolyte and separator, which not only increases the battery capacity but also promotes an effective charge transfer motion, thus leading to improved battery performance [28]. Generally speaking, a smaller contact angle means better wettability of the separator. Figure 4 displayed the photographs of the contact angle formed between the electrolyte of sodium-ion batteries and the surface of the separators (0.2 s after contact). It could be observed that all separators exhibited very small contact angles, with a maximum of 16.8° for the C/H-15 and a minimum of nearly 9.6° for the C/H-60. This was consistent with the results of electrolyte uptake in Table 1. The main reason was the special composition and structural features of the composite separators. In particular, the surfaces of the HNTs and CNFs within the separators were rich in polar functional groups, such as -OH and -COOH groups. These polar groups formed interactions such as hydrogen bonds with the electrolyte, enhancing the adsorption and retention of electrolyte by the composite separators [29,30]. In addition, the high porosity of the composite separator provided expansive channels for electrolyte infiltration. This allowed for the electrolyte to penetrate into the separator matrix quickly and efficiently, further improving the wettability.

### 3.4. Mechanical Properties of Composite Separators

**Figure 5:**
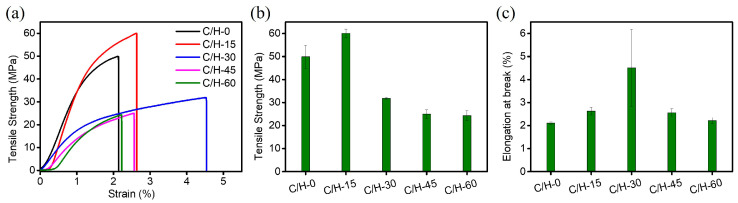
(**a**) Strain–tensile strength curves, (**b**) tensile strength, and (**c**) elongation at break of C/H-x composite separators.

Good mechanical properties of separators are essential to ensure the safety of batteries. In general, tensile strength and elongation at break are the main parameters for evaluating the mechanical properties of films. The related results were shown in Figure 5. As seen in Figure 5b, the tensile strength of C/H-0 film was 49.88 MPa; with the addition of different amounts of HNTs, the tensile strengths of the composite films were 60.06, 31.85, 25, and 24.39 MPa, respectively. The C/H-15 composite film showed the highest tensile strength, which may be attributed to the reinforcing effect of HNTs. The mechanical enhancement properties of nanofillers on composites are related to the effective transfer of load from the matrix to the nanofillers, which is achieved when there is a strong interaction at the interface between the nanofillers and the matrix, and the nanofillers are uniformly dispersed in the matrix [31]. There was an effective interaction between HNTs and BC nanofibers due to the presence of hydrogen bonds, which was conducive to dispersing external forces and avoiding stress concentration, and ultimately enhanced the mechanical properties of the composite film. However, with the increase in HNTs, accompanied by a decrease in CNF, agglomerates occurred, acting as defects in the film owing to excess HNTs, which caused the degraded tensile strength [32]. As shown in Figure 5c, the elongation at break value first increased and then decreased with the increase in HNT addition. The C/H-30 composite film displayed the highest elongation at break (4.51%), which was higher than that of the C/H-0 film (2.12%). At low HNT additions, HNTs disturbed the original regular arrangement of the CNF region and formed more microfiber chain slip, thus toughening the composite films. However, the elongation at break of the C/H-60 composite film decreased to 2.22%. Overdosed HNTs existed as aggregates in the CNF matrix, impeding chain movement and leading to a reduction in elongation at break.

### 3.5. Thermal Stability of Composite Separators

**Figure 6:**
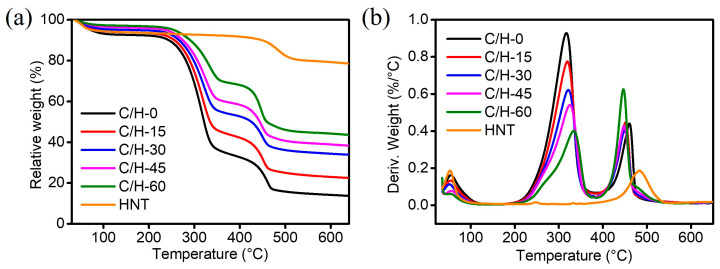
(**a**) TG and (**b**) DTG curves of C/H-x composite separators.

The thermal stability is a key factor in determining the properties of separators. If the thermal stability properties do not correspond to the actual temperature of use, it may cause serious safety accidents. The thermal stability of the samples was tested. The TG and DTG curves of the samples are shown in Figure 6. It could be seen that all samples kept a stable mass before 200 °C, with only a small weight loss attributed to water evaporation. HNTs exhibited high thermal stability, owing to their inorganic nature. For the C/H-0 separator, the first degradation occurred at approximately 250 °C, and the maximum weight loss rate happened at 317 °C. Notably, with the incorporation of HNTs, the onset temperature of the first degradation step of C/H-x composite separators was slightly higher. The results suggested that the HNT did not affect the thermal stability of the CNF-based separators.

### 3.6. Performance of Sodium-Ion Batteries with Different Composite Separators

**Figure 7:**
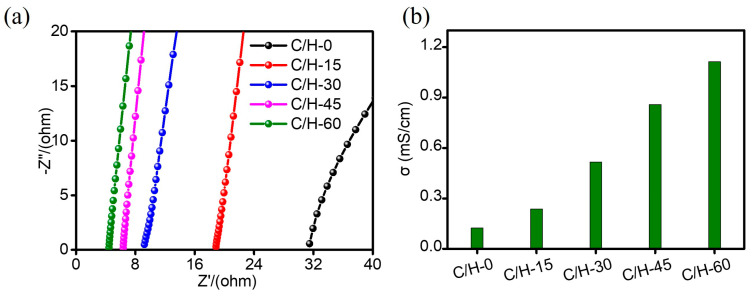
(**a**) EIS curves and (**b**) ionic conductivity of C/H-x composite separators.

For the purpose of obtaining bulk resistance (R_b_) and ionic conductivity of the separators, the EIS curves were measured by sandwiching the electrolyte-impregnated separator between two steel electrodes. The results are shown in Figure 7. The R_b_ was the intercept of the EIS spectrum at the real axis [33]. As seen in Table 1 and Figure 7b, the R_b_ and ionic conductivity of the C/H-0 separator were 31.48 Ω and 0.125 mS/cm, respectively. With the doping of HNTs, the R_b_ of the separators decreased while the ionic conductivity increased gradually. The C/H-60 separator presented the lowest resistance and highest ionic conductivity. This was attributed to the high porosity and good electrolyte retention capacity of the composite separators, which could promote the movement of ions and support the amount of ions migrating between the electrodes. It is important to note that the high porosity of the developed separator, while beneficial for electrolyte uptake and interfacial ion transport, necessitates a higher volume of electrolyte. This increases the inactive weight of the cell, presenting a trade-off against the overall weight energy density.

As the separators in the batteries will experience oxidative and reductive conditions during charge/discharge cycling, the separator should be electrochemically stable within the use window of the sodium-ion batteries. The electrochemical oxidation limit of the electrolyte-impregnated separator was determined using the LSV method (Figure 7a). In the LSV curve, a sharp increase in current can be noticed when the electrolyte starts to decompose [25]. The separators did not show anodic currents until the voltage was increased to 4.8 V. The results showed that the prepared separators possessed good electrochemical stability. Hence, it could be deduced that the composite separators were electrochemically inert at a voltage of up to 4.8 V.

**Figure 8:**
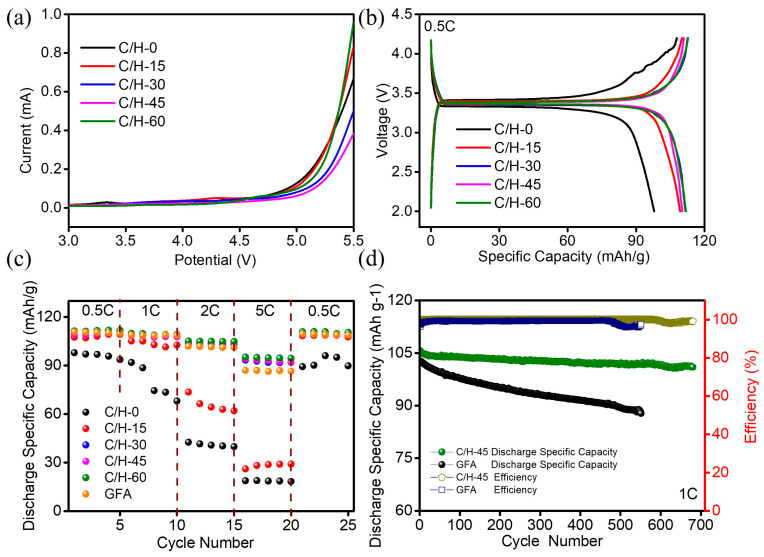
(**a**) LSV curves of C/H-x composite separators, (**b**) the first charge/discharge curves of C/H-x composite separators, (**c**) rate performance of separators, (**d**) cycle property of C/H-45 and GFA separators.

The capacities of batteries are primarily determined by the type and structure of the cathodes and anodes. However, studies have revealed that the type and structure of the separators also have a significant impact on the overall performance of the batteries, as the separators can affect the ion migration between the cathode and anode, which is important for monitoring the dynamics of the battery [34]. The charge/discharge curves of the batteries with different separators at 0.5 C were presented in Figure 8b, in which all the batteries using the separators had stable charge–discharge platforms. The discharge capacities of the batteries with composite separators were all higher than that of the battery with C/H-0 separator (97.9 mAh/g), which was primarily because of the superior porosity and ionic conductivity of the composite separators. The maximum discharge capacity of the battery using the C/H-60 separator was 111.4 mAh/g, around 94.73% of the theoretical specific capacity (117.6 mAh/g), which was better than that of the battery using the GFA separator (110.8 mAh/g).

The rate capabilities of the batteries with different separators at various current densities ranging from 0.5 C, 1 C, 2 C, 5 C, and 0.5 C are summarized in Figure 8c. It was clear that the battery with the C/H-60 separator exhibited better rate capability. For instance, the discharge capacity of the C/H-60 separator at 5 C was 95.2 mAh/g, about 85.46% of that at 0.5 C, while that for the C/H-0 separator was only around 19.31%. This was because the superior electrolyte wettability of the C/H-60 separator offered faster ionic transport, resulting in lower interfacial resistance, higher ionic conductivity, and better discharge rate capability. When the current density returned to 0.5 C, the discharge capacities of batteries with composite separators were almost completely back to the initial states, showing excellent reversibility. Batteries having C/H-45 and GFA separators were tested for cycling stability at a current density of 1C (Figure 8d). After 680 cycles, the battery with the C/H-45 separator kept 95.56% of its initial discharge capacity, in contrast to 85.57% for the battery with the GFA separator after 551 cycles. In addition, the efficiency was close to 99% even after 680 cycles for the battery with C/H-45 separator, which demonstrated that the battery with C/H-45 separator possessed better cycling performance. The improved cycling performance was correlated with exceptional wettability, high electrolyte uptake, and favorable interfacial compatibility.

## 4. Conclusions

In this paper, a series of composite films used as sodium-ion battery separators was prepared by combining CNFs and HNTs through a simple vacuum filtration process and paper-making method. The addition of HNT significantly affected the pore structure of the separators, with the highest porosity reaching 69.08%, which exceeded that of the pure CNF-based separator (33.61%). Thanks to the superior pore structure and the presence of polar groups on the surface, the C/H-x composite separators exhibited excellent wetting of the electrolyte used in the sodium-ion batteries, with a contact angle close to 9.6°. The electrolyte uptake and ionic conductivity of the C/H-60 separator reached 91.30% and 1.142 mS/cm, respectively. The TG testing revealed that the composite separators had good thermal stability. Moreover, along with the increase in HNT content in the composite separators, the tensile strength and elongation at break showed a trend of first increasing and then decreasing. The sodium-ion battery using the C/H-60 separator showed excellent rate capability, with a discharge specific capacity of 111.4 mAh/g at 0.5 C. After 680 charge/discharge cycles for the sodium-ion battery with the C/H-45 separator, the capacity retention remained at 95.56%, with an efficiency over 99%. These results demonstrate that the C/H-x composite separators would be promising candidates for use in high-performance sodium-ion batteries.

## Figures and Tables

**Table 1:** The properties of the C/H-x composite separators.

Samples	C/H-0	C/H-15	C/H-30	C/H-45	C/H-60
Mass ratio of HNT (%)	0	15	30	45	60
Thickness (mm)	0.078	0.089	0.094	0.108	0.100
Porosity (%)	33.61	41.84	45.03	53.58	69.08
Electrolyte uptake (%)	42.74	49.14	63.64	82.67	91.30
Bulk resistance (R_b_, Ω)	31.48	18.88	9.213	6.353	4.425

## Data Availability

Data will be made available on request.
